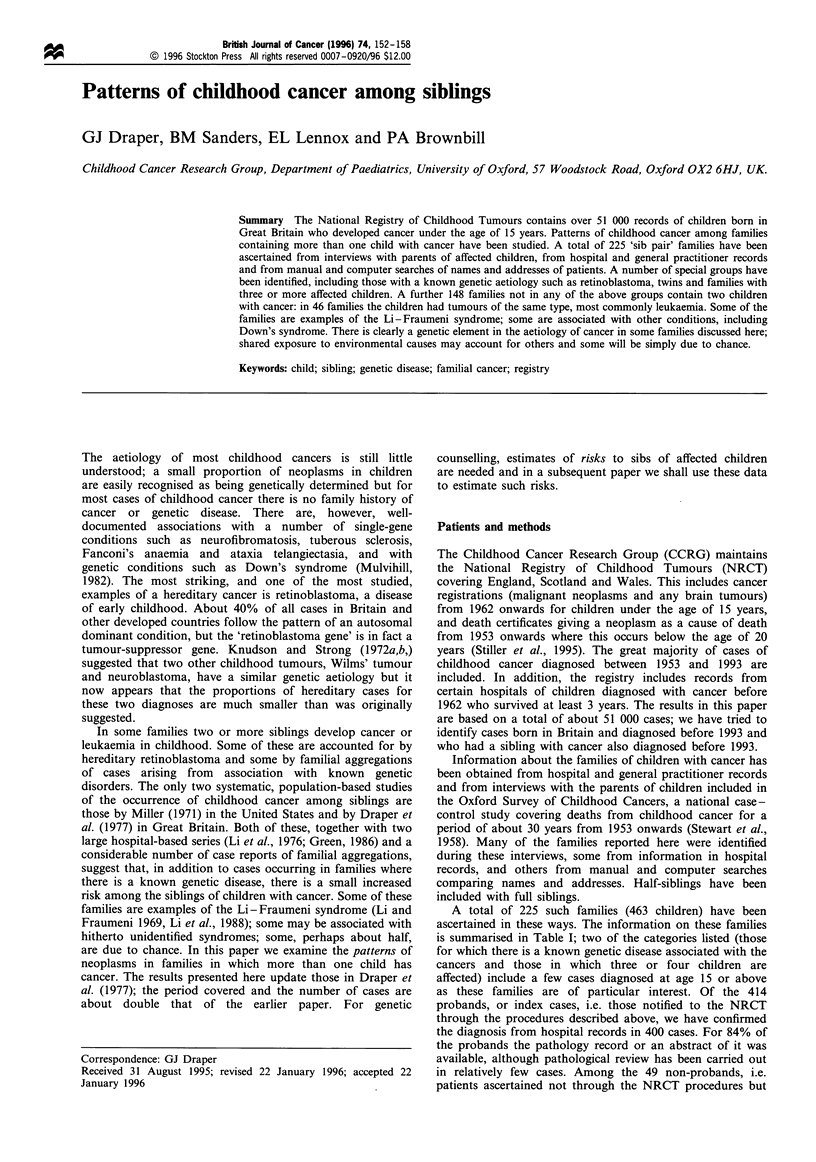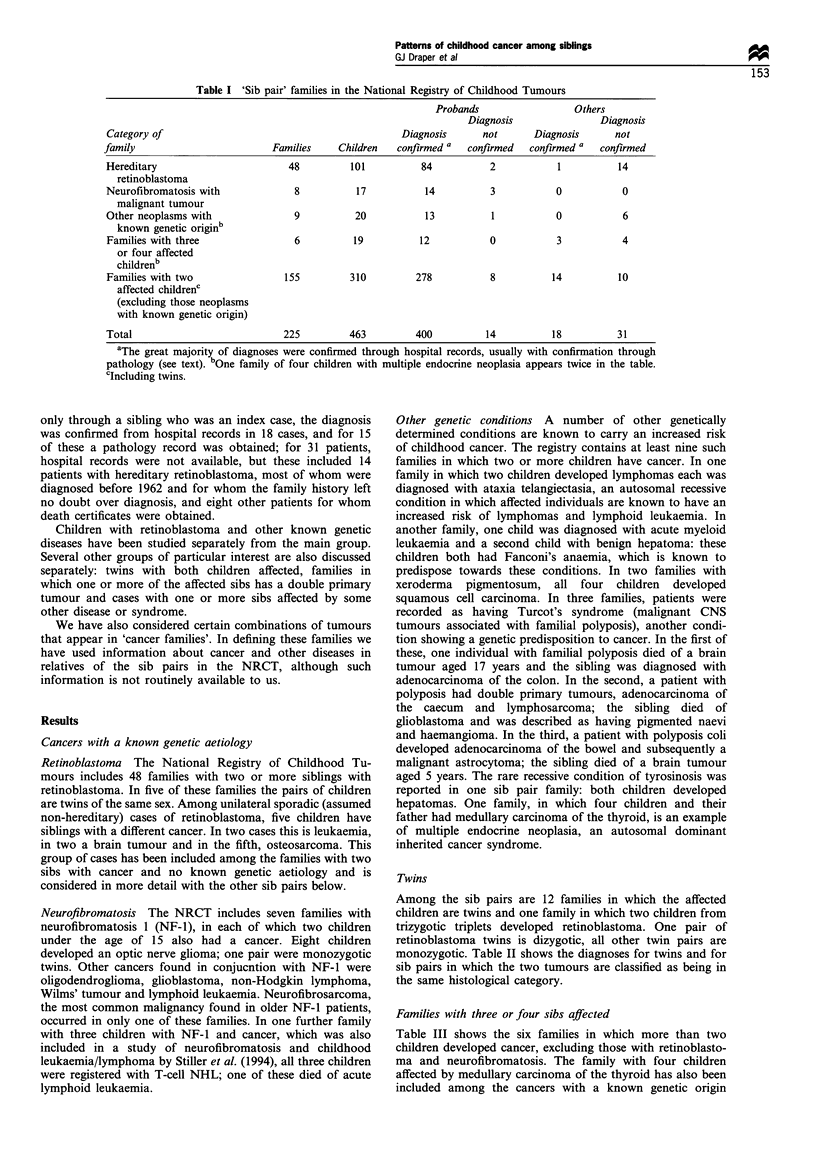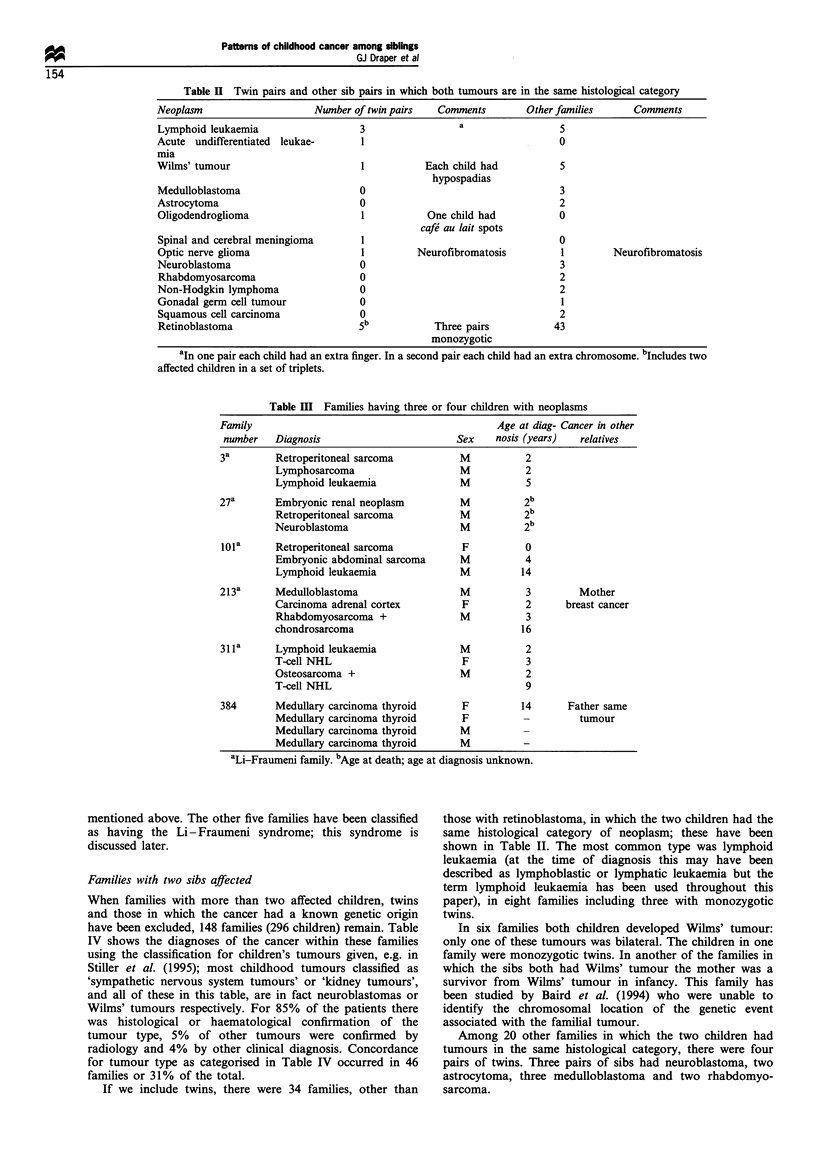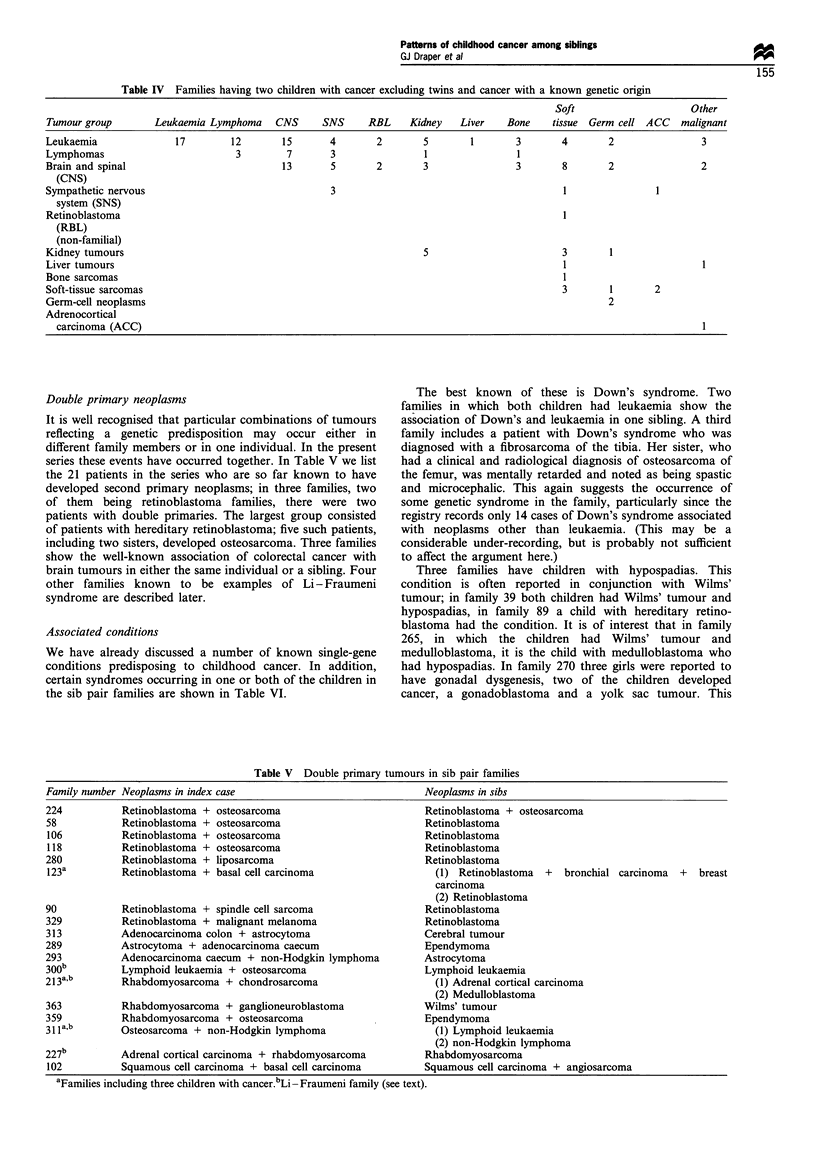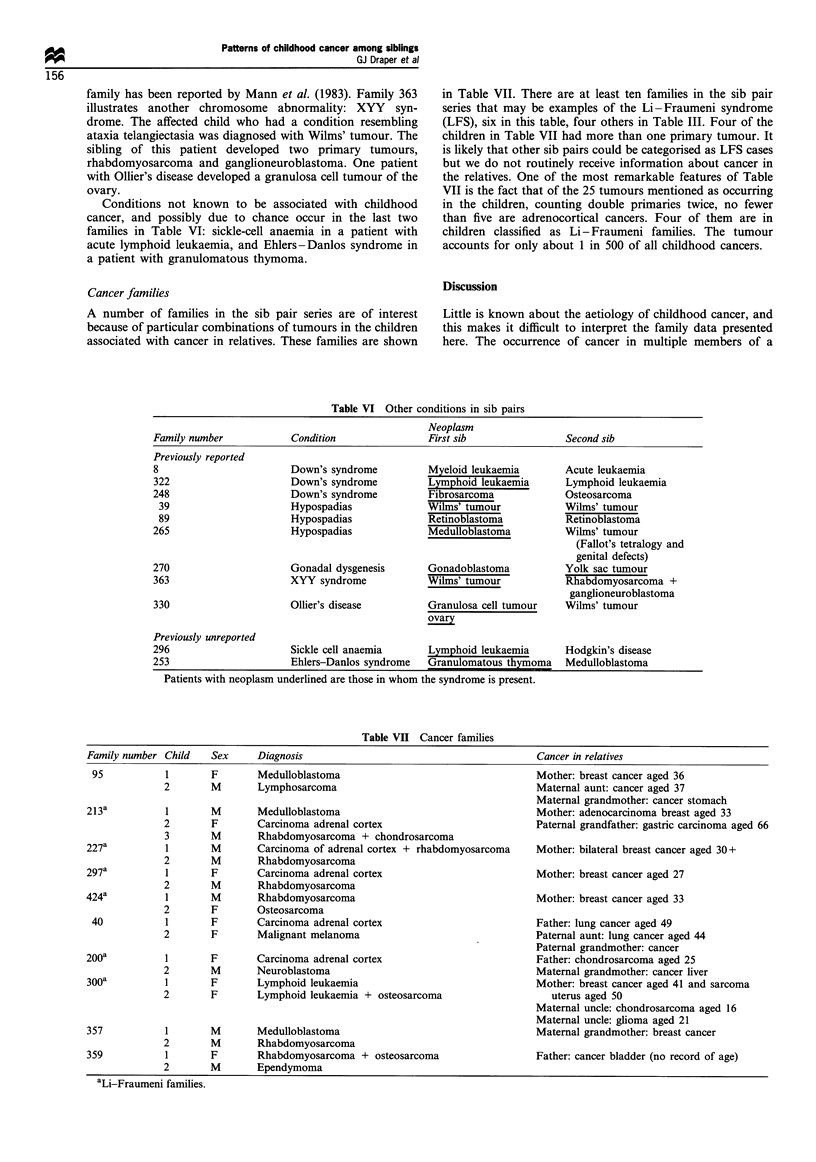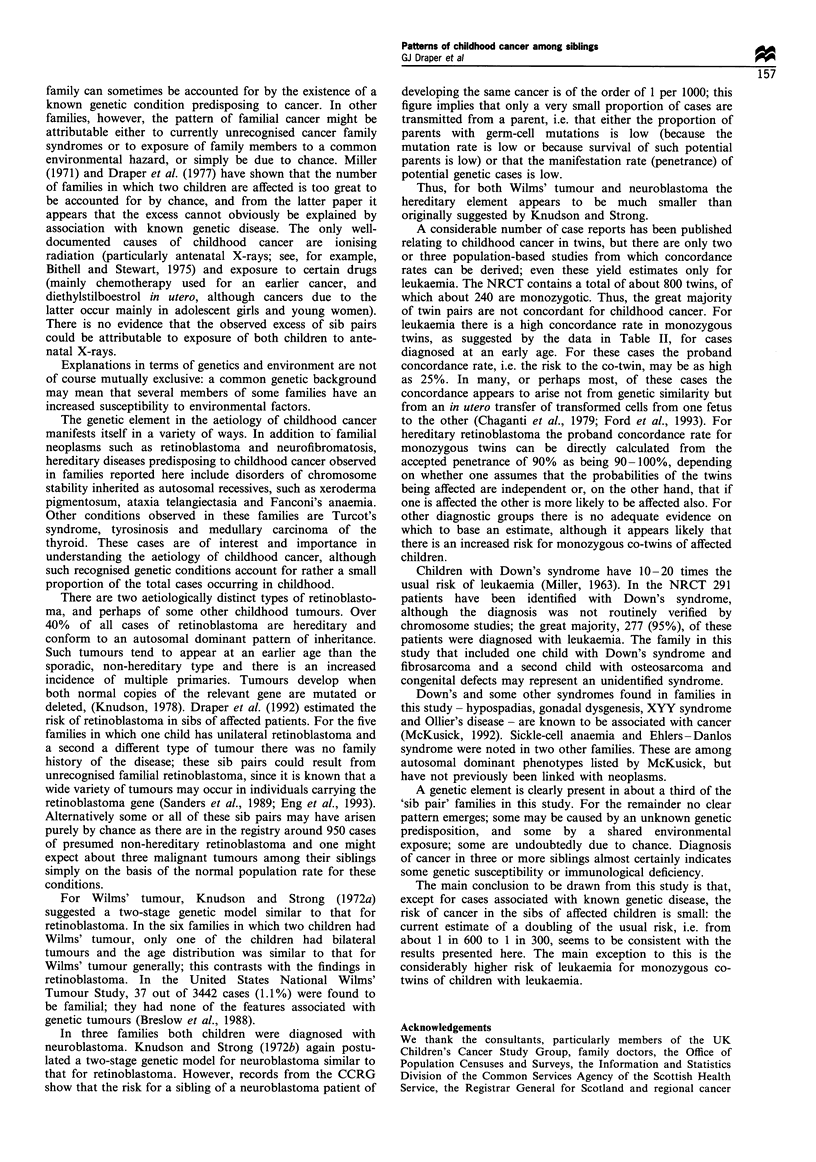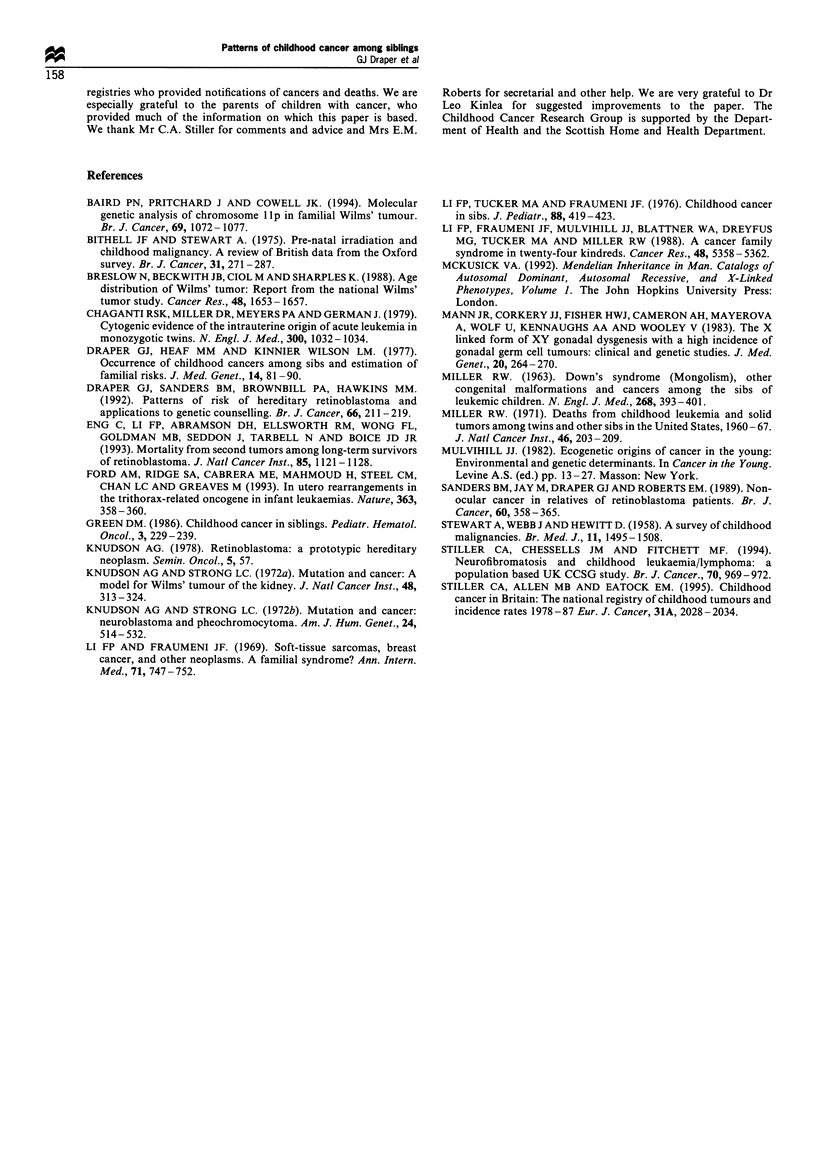# Patterns of childhood cancer among siblings.

**DOI:** 10.1038/bjc.1996.331

**Published:** 1996-07

**Authors:** G. J. Draper, B. M. Sanders, E. L. Lennox, P. A. Brownbill

**Affiliations:** Department of Paediatrics, University of Oxford, UK.

## Abstract

The National Registry of Childhood Tumours contains over 51000 records of children born in Great Britain who developed cancer under the age of 15 years. Patterns of childhood cancer among families containing more than one child with cancer have been studied. A total of 225 "sib pair' families have been ascertained from interviews with parents of affected children, from hospital and general practitioner records and from manual and computer searches of names and addresses of patients. A number of special groups have been identified, including those with a known genetic aetiology such as retinoblastoma, twins and families with three or more affected children. A further 148 families not in any of the above groups contain two children with cancer: in 46 families the children had tumours of the same type, most commonly leukaemia. Some of the families are examples of the Li-Fraumeni syndrome; some are associated with other conditions, including Down's syndrome. There is clearly a genetic element in the aetiology of cancer in some families discussed here; shared exposure to environmental causes may account for others and some will be simply due to chance.


British Journal of Cancer (1996) 74, 152-158
© 1996 Stockton Press All rights reserved 0007-0920/96 $12.00

### Patterns of childhood cancer among siblings

GJ Draper, BM Sanders, EL Lennox and PA Brownbill

*Childhood Cancer Research Group, Department of Paediatrics, University of Oxford, 57 Woodstock Road, Oxford OX2 6HJ, UK.*

**Summary** The National Registry of Childhood Tumours contains over 51 000 records of children born in Great Britain who developed cancer under the age of 15 years. Patterns of childhood cancer among families containing more than one child with cancer have been studied. A total of 225 'sib pair' families have been ascertained from interviews with parents of affected children, from hospital and general practitioner records and from manual and computer searches of names and addresses of patients. A number of special groups have been identified, including those with a known genetic aetiology such as retinoblastoma, twins and families with three or more affected children. A further 148 families not in any of the above groups contain two children with cancer: in 46 families the children had tumours of the same type, most commonly leukaemia. Some of the families are examples of the Li–Fraumeni syndrome; some are associated with other conditions, including Down's syndrome. There is clearly a genetic element in the aetiology of cancer in some families discussed here; shared exposure to environmental causes may account for others and some will be simply due to chance.

**Keywords:** child; sibling; genetic disease; familial cancer; registry

The aetiology of most childhood cancers is still little understood; a small proportion of neoplasms in children are easily recognised as being genetically determined but for most cases of childhood cancer there is no family history of cancer or genetic disease. There are, however, well-documented associations with a number of single-gene conditions such as neurofibromatosis, tuberous sclerosis, Fanconi's anaemia and ataxia telangiectasia, and with genetic conditions such as Down's syndrome (Mulvihill, 1982). The most striking, and one of the most studied, examples of a hereditary cancer is retinoblastoma, a disease of early childhood. About 40% of all cases in Britain and other developed countries follow the pattern of an autosomal dominant condition, but the 'retinoblastoma gene' is in fact a tumour-suppressor gene. Knudson and Strong (1972*a*,*b*,) suggested that two other childhood tumours, Wilms' tumour and neuroblastoma, have a similar genetic aetiology but it now appears that the proportions of hereditary cases for these two diagnoses are much smaller than was originally suggested.

In some families two or more siblings develop cancer or leukaemia in childhood. Some of these are accounted for by hereditary retinoblastoma and some by familial aggregations of cases arising from association with known genetic disorders. The only two systematic, population-based studies of the occurrence of childhood cancer among siblings are those by Miller (1971) in the United States and by Draper *et al.* (1977) in Great Britain. Both of these, together with two large hospital-based series (Li *et al.*, 1976; Green, 1986) and a considerable number of case reports of familial aggregations, suggest that, in addition to cases occurring in families where there is a known genetic disease, there is a small increased risk among the siblings of children with cancer. Some of these families are examples of the Li–Fraumeni syndrome (Li and Fraumeni 1969, Li *et al.*, 1988); some may be associated with hitherto unidentified syndromes; some, perhaps about half, are due to chance. In this paper we examine the *patterns* of neoplasms in families in which more than one child has cancer. The results presented here update those in Draper *et al.* (1977); the period covered and the number of cases are about double that of the earlier paper. For genetic counselling, estimates of *risks* to sibs of affected children are needed and in a subsequent paper we shall use these data to estimate such risks.

Correspondence: GJ Draper
Received 31 August 1995; revised 22 January 1996; accepted 22 January 1996

### Patients and methods

The Childhood Cancer Research Group (CCRG) maintains the National Registry of Childhood Tumours (NRCT) covering England, Scotland and Wales. This includes cancer registrations (malignant neoplasms and any brain tumours) from 1962 onwards for children under the age of 15 years, and death certificates giving a neoplasm as a cause of death from 1953 onwards where this occurs below the age of 20 years (Stiller *et al.*, 1995). The great majority of cases of childhood cancer diagnosed between 1953 and 1993 are included. In addition, the registry includes records from certain hospitals of children diagnosed with cancer before 1962 who survived at least 3 years. The results in this paper are based on a total of about 51 000 cases; we have tried to identify cases born in Britain and diagnosed before 1993 and who had a sibling with cancer also diagnosed before 1993.

Information about the families of children with cancer has been obtained from hospital and general practitioner records and from interviews with the parents of children included in the Oxford Survey of Childhood Cancers, a national case–control study covering deaths from childhood cancer for a period of about 30 years from 1953 onwards (Stewart *et al.*, 1958). Many of the families reported here were identified during these interviews, some from information in hospital records, and others from manual and computer searches comparing names and addresses. Half-siblings have been included with full siblings.

A total of 225 such families (463 children) have been ascertained in these ways. The information on these families is summarised in Table I; two of the categories listed (those for which there is a known genetic disease associated with the cancers and those in which three or four children are affected) include a few cases diagnosed at age 15 or above as these families are of particular interest. Of the 414 probands, or index cases, i.e. those notified to the NRCT through the procedures described above, we have confirmed the diagnosis from hospital records in 400 cases. For 84% of the probands the pathology record or an abstract of it was available, although pathological review has been carried out in relatively few cases. Among the 49 non-probands, i.e. patients ascertained not through the NRCT procedures but

**Table I** 'Sib pair' families in the National Registry of Childhood Tumours

| *Category of family* | *Families* | *Children* | *Probands* | | *Others* | |
|---|---|---|---|---|---|---|
| | | | *Diagnosis confirmed* [a] | *Diagnosis not confirmed* | *Diagnosis confirmed* [a] | *Diagnosis not confirmed* |
| Hereditary retinoblastoma | 48 | 101 | 84 | 2 | 1 | 14 |
| Neurofibromatosis with malignant tumour | 8 | 17 | 14 | 3 | 0 | 0 |
| Other neoplasms with known genetic origin[b] | 9 | 20 | 13 | 1 | 0 | 6 |
| Families with three or four affected children[b] | 6 | 19 | 12 | 0 | 3 | 4 |
| Families with two affected children[c] (excluding those neoplasms with known genetic origin) | 155 | 310 | 278 | 8 | 14 | 10 |
| Total | 225 | 463 | 400 | 14 | 18 | 31 |

[a]The great majority of diagnoses were confirmed through hospital records, usually with confirmation through pathology (see text). [b]One family of four children with multiple endocrine neoplasia appears twice in the table. [c]Including twins.

only through a sibling who was an index case, the diagnosis was confirmed from hospital records in 18 cases, and for 15 of these a pathology record was obtained; for 31 patients, hospital records were not available, but these included 14 patients with hereditary retinoblastoma, most of whom were diagnosed before 1962 and for whom the family history left no doubt over diagnosis, and eight other patients for whom death certificates were obtained.

Children with retinoblastoma and other known genetic diseases have been studied separately from the main group. Several other groups of particular interest are also discussed separately: twins with both children affected, families in which one or more of the affected sibs has a double primary tumour and cases with one or more sibs affected by some other disease or syndrome.

We have also considered certain combinations of tumours that appear in 'cancer families'. In defining these families we have used information about cancer and other diseases in relatives of the sib pairs in the NRCT, although such information is not routinely available to us.

### Results

### *Cancers with a known genetic aetiology*

*Retinoblastoma* The National Registry of Childhood Tumours includes 48 families with two or more siblings with retinoblastoma. In five of these families the pairs of children are twins of the same sex. Among unilateral sporadic (assumed non-hereditary) cases of retinoblastoma, five children have siblings with a different cancer. In two cases this is leukaemia, in two a brain tumour and in the fifth, osteosarcoma. This group of cases has been included among the families with two sibs with cancer and no known genetic aetiology and is considered in more detail with the other sib pairs below.

*Neurofibromatosis* The NRCT includes seven families with neurofibromatosis 1 (NF-1), in each of which two children under the age of 15 also had a cancer. Eight children developed an optic nerve glioma; one pair were monozygotic twins. Other cancers found in conjucntion with NF-1 were oligodendroglioma, glioblastoma, non-Hodgkin lymphoma, Wilms' tumour and lymphoid leukaemia. Neurofibrosarcoma, the most common malignancy found in older NF-1 patients, occurred in only one of these families. In one further family with three children with NF-1 and cancer, which was also included in a study of neurofibromatosis and childhood leukaemia/lymphoma by Stiller *et al.* (1994), all three children were registered with T-cell NHL; one of these died of acute lymphoid leukaemia.

*Other genetic conditions* A number of other genetically determined conditions are known to carry an increased risk of childhood cancer. The registry contains at least nine such families in which two or more children have cancer. In one family in which two children developed lymphomas each was diagnosed with ataxia telangiectasia, an autosomal recessive condition in which affected individuals are known to have an increased risk of lymphomas and lymphoid leukaemia. In another family, one child was diagnosed with acute myeloid leukaemia and a second child with benign hepatoma: these children both had Fanconi's anaemia, which is known to predispose towards these conditions. In two families with xeroderma pigmentosum, all four children developed squamous cell carcinoma. In three families, patients were recorded as having Turcot's syndrome (malignant CNS tumours associated with familial polyposis), another condition showing a genetic predisposition to cancer. In the first of these, one individual with familial polyposis died of a brain tumour aged 17 years and the sibling was diagnosed with adenocarcinoma of the colon. In the second, a patient with polyposis had double primary tumours, adenocarcinoma of the caecum and lymphosarcoma; the sibling died of glioblastoma and was described as having pigmented naevi and haemangioma. In the third, a patient with polyposis coli developed adenocarcinoma of the bowel and subsequently a malignant astrocytoma; the sibling died of a brain tumour aged 5 years. The rare recessive condition of tyrosinosis was reported in one sib pair family: both children developed hepatomas. One family, in which four children and their father had medullary carcinoma of the thyroid, is an example of multiple endocrine neoplasia, an autosomal dominant inherited cancer syndrome.

### *Twins*

Among the sib pairs are 12 families in which the affected children are twins and one family in which two children from trizygotic triplets developed retinoblastoma. One pair of retinoblastoma twins is dizygotic, all other twin pairs are monozygotic. Table II shows the diagnoses for twins and for sib pairs in which the two tumours are classified as being in the same histological category.

### *Families with three or four sibs affected*

Table III shows the six families in which more than two children developed cancer, excluding those with retinoblastoma and neurofibromatosis. The family with four children affected by medullary carcinoma of the thyroid has also been included among the cancers with a known genetic origin

**Table II** Twin pairs and other sib pairs in which both tumours are in the same histological category

| *Neoplasm* | *Number of twin pairs* | *Comments* | *Other families* | *Comments* |
|---|---|---|---|---|
| Lymphoid leukaemia | 3 | a | 5 | |
| Acute undifferentiated leukaemia | 1 | | 0 | |
| Wilms' tumour | 1 | Each child had hypospadias | 5 | |
| Medulloblastoma | 0 | | 3 | |
| Astrocytoma | 0 | | 2 | |
| Oligodendroglioma | 1 | One child had *café au lait* spots | 0 | |
| Spinal and cerebral meningioma | 1 | | 0 | |
| Optic nerve glioma | 1 | Neurofibromatosis | 1 | Neurofibromatosis |
| Neuroblastoma | 0 | | 3 | |
| Rhabdomyosarcoma | 0 | | 2 | |
| Non-Hodgkin lymphoma | 0 | | 2 | |
| Gonadal germ cell tumour | 0 | | 1 | |
| Squamous cell carcinoma | 0 | | 2 | |
| Retinoblastoma | 5[b] | Three pairs monozygotic | 43 | |

[a]In one pair each child had an extra finger. In a second pair each child had an extra chromosome. [b]Includes two affected children in a set of triplets.

**Table III** Families having three or four children with neoplasms

| *Family number* | *Diagnosis* | *Sex* | *Age at diagnosis (years)* | *Cancer in other relatives* |
|---|---|---|---|---|
| 3[a] | Retroperitoneal sarcoma | M | 2 | |
| | Lymphosarcoma | M | 2 | |
| | Lymphoid leukaemia | M | 5 | |
| 27[a] | Embryonic renal neoplasm | M | 2[b] | |
| | Retroperitoneal sarcoma | M | 2[b] | |
| | Neuroblastoma | M | 2[b] | |
| 101[a] | Retroperitoneal sarcoma | F | 0 | |
| | Embryonic abdominal sarcoma | M | 4 | |
| | Lymphoid leukaemia | M | 14 | |
| 213[a] | Medulloblastoma | M | 3 | Mother |
| | Carcinoma adrenal cortex | F | 2 | breast cancer |
| | Rhabdomyosarcoma + | M | 3 | |
| | chondrosarcoma | | 16 | |
| 311[a] | Lymphoid leukaemia | M | 2 | |
| | T-cell NHL | F | 3 | |
| | Osteosarcoma + | M | 2 | |
| | T-cell NHL | | 9 | |
| 384 | Medullary carcinoma thyroid | F | 14 | Father same |
| | Medullary carcinoma thyroid | F | – | tumour |
| | Medullary carcinoma thyroid | M | – | |
| | Medullary carcinoma thyroid | M | – | |

[a]Li–Fraumeni family. [b]Age at death; age at diagnosis unknown.

mentioned above. The other five families have been classified as having the Li–Fraumeni syndrome; this syndrome is discussed later.

*Families with two sibs affected*

When families with more than two affected children, twins and those in which the cancer had a known genetic origin have been excluded, 148 families (296 children) remain. Table IV shows the diagnoses of the cancer within these families using the classification for children's tumours given, e.g. in Stiller *et al.* (1995); most childhood tumours classified as 'sympathetic nervous system tumours' or 'kidney tumours', and all of these in this table, are in fact neuroblastomas or Wilms' tumours respectively. For 85% of the patients there was histological or haematological confirmation of the tumour type, 5% of other tumours were confirmed by radiology and 4% by other clinical diagnosis. Concordance for tumour type as categorised in Table IV occurred in 46 families or 31% of the total.

If we include twins, there were 34 families, other than those with retinoblastoma, in which the two children had the same histological category of neoplasm; these have been shown in Table II. The most common type was lymphoid leukaemia (at the time of diagnosis this may have been described as lymphoblastic or lymphatic leukaemia but the term lymphoid leukaemia has been used throughout this paper), in eight families including three with monozygotic twins.

In six families both children developed Wilms' tumour: only one of these tumours was bilateral. The children in one family were monozygotic twins. In another of the families in which the sibs both had Wilms' tumour the mother was a survivor from Wilms' tumour in infancy. This family has been studied by Baird *et al.* (1994) who were unable to identify the chromosomal location of the genetic event associated with the familial tumour.

Among 20 other families in which the two children had tumours in the same histological category, there were four pairs of twins. Three pairs of sibs had neuroblastoma, two astrocytoma, three medulloblastoma and two rhabdomyosarcoma.

**Table IV** Families having two children with cancer excluding twins and cancer with a known genetic origin

| *Tumour group* | *Leukaemia* | *Lymphoma* | *CNS* | *SNS* | *RBL* | *Kidney* | *Liver* | *Bone* | *Soft tissue* | *Germ cell* | *ACC* | *Other malignant* |
|---|---|---|---|---|---|---|---|---|---|---|---|---|
| Leukaemia | 17 | 12 | 15 | 4 | 2 | 5 | 1 | 3 | 4 | 2 | | 3 |
| Lymphomas | | 3 | 7 | 3 | | 1 | | 1 | | | | |
| Brain and spinal (CNS) | | | 13 | 5 | 2 | 3 | | 3 | 8 | 2 | | 2 |
| Sympathetic nervous system (SNS) | | | | 3 | | | | | 1 | | 1 | |
| Retinoblastoma (RBL) (non-familial) | | | | | | | | | 1 | | | |
| Kidney tumours | | | | | | 5 | | | 3 | 1 | | |
| Liver tumours | | | | | | | | | 1 | | | 1 |
| Bone sarcomas | | | | | | | | | 1 | | | |
| Soft-tissue sarcomas | | | | | | | | | 3 | 1 | 2 | |
| Germ-cell neoplasms | | | | | | | | | | 2 | | |
| Adrenocortical carcinoma (ACC) | | | | | | | | | | | | 1 |

*Double primary neoplasms*

It is well recognised that particular combinations of tumours reflecting a genetic predisposition may occur either in different family members or in one individual. In the present series these events have occurred together. In Table V we list the 21 patients in the series who are so far known to have developed second primary neoplasms; in three families, two of them being retinoblastoma families, there were two patients with double primaries. The largest group consisted of patients with hereditary retinoblastoma; five such patients, including two sisters, developed osteosarcoma. Three families show the well-known association of colorectal cancer with brain tumours in either the same individual or a sibling. Four other families known to be examples of Li–Fraumeni syndrome are described later.

*Associated conditions*

We have already discussed a number of known single-gene conditions predisposing to childhood cancer. In addition, certain syndromes occurring in one or both of the children in the sib pair families are shown in Table VI.

The best known of these is Down's syndrome. Two families in which both children had leukaemia show the association of Down's and leukaemia in one sibling. A third family includes a patient with Down's syndrome who was diagnosed with a fibrosarcoma of the tibia. Her sister, who had a clinical and radiological diagnosis of osteosarcoma of the femur, was mentally retarded and noted as being spastic and microcephalic. This again suggests the occurrence of some genetic syndrome in the family, particularly since the registry records only 14 cases of Down's syndrome associated with neoplasms other than leukaemia. (This may be a considerable under-recording, but is probably not sufficient to affect the argument here.)

Three families have children with hypospadias. This condition is often reported in conjunction with Wilms' tumour; in family 39 both children had Wilms' tumour and hypospadias, in family 89 a child with hereditary retinoblastoma had the condition. It is of interest that in family 265, in which the children had Wilms' tumour and medulloblastoma, it is the child with medulloblastoma who had hypospadias. In family 270 three girls were reported to have gonadal dysgenesis, two of the children developed cancer, a gonadoblastoma and a yolk sac tumour. This

**Table V** Double primary tumours in sib pair families

| *Family number* | *Neoplasms in index case* | *Neoplasms in sibs* |
|---|---|---|
| 224 | Retinoblastoma + osteosarcoma | Retinoblastoma + osteosarcoma |
| 58 | Retinoblastoma + osteosarcoma | Retinoblastoma |
| 106 | Retinoblastoma + osteosarcoma | Retinoblastoma |
| 118 | Retinoblastoma + osteosarcoma | Retinoblastoma |
| 280 | Retinoblastoma + liposarcoma | Retinoblastoma |
| 123[a] | Retinoblastoma + basal cell carcinoma | (1) Retinoblastoma + bronchial carcinoma + breast carcinoma<br>(2) Retinoblastoma |
| 90 | Retinoblastoma + spindle cell sarcoma | Retinoblastoma |
| 329 | Retinoblastoma + malignant melanoma | Retinoblastoma |
| 313 | Adenocarcinoma colon + astrocytoma | Cerebral tumour |
| 289 | Astrocytoma + adenocarcinoma caecum | Ependymoma |
| 293 | Adenocarcinoma caecum + non-Hodgkin lymphoma | Astrocytoma |
| 300[b] | Lymphoid leukaemia + osteosarcoma | Lymphoid leukaemia |
| 213[a,b] | Rhabdomyosarcoma + chondrosarcoma | (1) Adrenal cortical carcinoma<br>(2) Medulloblastoma |
| 363 | Rhabdomyosarcoma + ganglioneuroblastoma | Wilms' tumour |
| 359 | Rhabdomyosarcoma + osteosarcoma | Ependymoma |
| 311[a,b] | Osteosarcoma + non-Hodgkin lymphoma | (1) Lymphoid leukaemia<br>(2) non-Hodgkin lymphoma |
| 227[b] | Adrenal cortical carcinoma + rhabdomyosarcoma | Rhabdomyosarcoma |
| 102 | Squamous cell carcinoma + basal cell carcinoma | Squamous cell carcinoma + angiosarcoma |

[a]Families including three children with cancer.[b]Li–Fraumeni family (see text).

family has been reported by Mann *et al.* (1983). Family 363 illustrates another chromosome abnormality: XYY syndrome. The affected child who had a condition resembling ataxia telangiectasia was diagnosed with Wilms' tumour. The sibling of this patient developed two primary tumours, rhabdomyosarcoma and ganglioneuroblastoma. One patient with Ollier's disease developed a granulosa cell tumour of the ovary.

Conditions not known to be associated with childhood cancer, and possibly due to chance occur in the last two families in Table VI: sickle-cell anaemia in a patient with acute lymphoid leukaemia, and Ehlers–Danlos syndrome in a patient with granulomatous thymoma.

### *Cancer families*

A number of families in the sib pair series are of interest because of particular combinations of tumours in the children associated with cancer in relatives. These families are shown in Table VII. There are at least ten families in the sib pair series that may be examples of the Li–Fraumeni syndrome (LFS), six in this table, four others in Table III. Four of the children in Table VII had more than one primary tumour. It is likely that other sib pairs could be categorised as LFS cases but we do not routinely receive information about cancer in the relatives. One of the most remarkable features of Table VII is the fact that of the 25 tumours mentioned as occurring in the children, counting double primaries twice, no fewer than five are adrenocortical cancers. Four of them are in children classified as Li–Fraumeni families. The tumour accounts for only about 1 in 500 of all childhood cancers.

### Discussion

Little is known about the aetiology of childhood cancer, and this makes it difficult to interpret the family data presented here. The occurrence of cancer in multiple members of a

**Table VI** Other conditions in sib pairs

| *Family number* | *Condition* | *Neoplasm* *First sib* | *Second sib* |
|---|---|---|---|
| *Previously reported* | | | |
| 8 | Down's syndrome | Myeloid leukaemia | Acute leukaemia |
| 322 | Down's syndrome | Lymphoid leukaemia | Lymphoid leukaemia |
| 248 | Down's syndrome | Fibrosarcoma | Osteosarcoma |
| 39 | Hypospadias | Wilms' tumour | Wilms' tumour |
| 89 | Hypospadias | Retinoblastoma | Retinoblastoma |
| 265 | Hypospadias | Medulloblastoma | Wilms' tumour (Fallot's tetralogy and genital defects) |
| 270 | Gonadal dysgenesis | Gonadoblastoma | Yolk sac tumour |
| 363 | XYY syndrome | Wilms' tumour | Rhabdomyosarcoma + ganglioneuroblastoma |
| 330 | Ollier's disease | Granulosa cell tumour ovary | Wilms' tumour |
| *Previously unreported* | | | |
| 296 | Sickle cell anaemia | Lymphoid leukaemia | Hodgkin's disease |
| 253 | Ehlers–Danlos syndrome | Granulomatous thymoma | Medulloblastoma |

Patients with neoplasm underlined are those in whom the syndrome is present.

**Table VII** Cancer families

| *Family number* | *Child* | *Sex* | *Diagnosis* | *Cancer in relatives* |
|---|---|---|---|---|
| 95 | 1 | F | Medulloblastoma | Mother: breast cancer aged 36 |
| | 2 | M | Lymphosarcoma | Maternal aunt: cancer aged 37 |
| | | | | Maternal grandmother: cancer stomach |
| 213[a] | 1 | M | Medulloblastoma | Mother: adenocarcinoma breast aged 33 |
| | 2 | F | Carcinoma adrenal cortex | Paternal grandfather: gastric carcinoma aged 66 |
| | 3 | M | Rhabdomyosarcoma + chondrosarcoma | |
| 227[a] | 1 | M | Carcinoma of adrenal cortex + rhabdomyosarcoma | Mother: bilateral breast cancer aged 30+ |
| | 2 | M | Rhabdomyosarcoma | |
| 297[a] | 1 | F | Carcinoma adrenal cortex | Mother: breast cancer aged 27 |
| | 2 | M | Rhabdomyosarcoma | |
| 424[a] | 1 | M | Rhabdomyosarcoma | Mother: breast cancer aged 33 |
| | 2 | F | Osteosarcoma | |
| 40 | 1 | F | Carcinoma adrenal cortex | Father: lung cancer aged 49 |
| | 2 | F | Malignant melanoma | Paternal aunt: lung cancer aged 44 |
| | | | | Paternal grandmother: cancer |
| 200[a] | 1 | F | Carcinoma adrenal cortex | Father: chondrosarcoma aged 25 |
| | 2 | M | Neuroblastoma | Maternal grandmother: cancer liver |
| 300[a] | 1 | F | Lymphoid leukaemia | Mother: breast cancer aged 41 and sarcoma uterus aged 50 |
| | 2 | F | Lymphoid leukaemia + osteosarcoma | |
| | | | | Maternal uncle: chondrosarcoma aged 16 |
| | | | | Maternal uncle: glioma aged 21 |
| 357 | 1 | M | Medulloblastoma | Maternal grandmother: breast cancer |
| | 2 | M | Rhabdomyosarcoma | |
| 359 | 1 | F | Rhabdomyosarcoma + osteosarcoma | Father: cancer bladder (no record of age) |
| | 2 | M | Ependymoma | |

[a]Li–Fraumeni families.

family can sometimes be accounted for by the existence of a known genetic condition predisposing to cancer. In other families, however, the pattern of familial cancer might be attributable either to currently unrecognised cancer family syndromes or to exposure of family members to a common environmental hazard, or simply be due to chance. Miller (1971) and Draper *et al.* (1977) have shown that the number of families in which two children are affected is too great to be accounted for by chance, and from the latter paper it appears that the excess cannot obviously be explained by association with known genetic disease. The only well-documented causes of childhood cancer are ionising radiation (particularly antenatal X-rays; see, for example, Bithell and Stewart, 1975) and exposure to certain drugs (mainly chemotherapy used for an earlier cancer, and diethylstilboestrol *in utero*, although cancers due to the latter occur mainly in adolescent girls and young women). There is no evidence that the observed excess of sib pairs could be attributable to exposure of both children to antenatal X-rays.

Explanations in terms of genetics and environment are not of course mutually exclusive: a common genetic background may mean that several members of some families have an increased susceptibility to environmental factors.

The genetic element in the aetiology of childhood cancer manifests itself in a variety of ways. In addition to familial neoplasms such as retinoblastoma and neurofibromatosis, hereditary diseases predisposing to childhood cancer observed in families reported here include disorders of chromosome stability inherited as autosomal recessives, such as xeroderma pigmentosum, ataxia telangiectasia and Fanconi's anaemia. Other conditions observed in these families are Turcot's syndrome, tyrosinosis and medullary carcinoma of the thyroid. These cases are of interest and importance in understanding the aetiology of childhood cancer, although such recognised genetic conditions account for rather a small proportion of the total cases occurring in childhood.

There are two aetiologically distinct types of retinoblastoma, and perhaps of some other childhood tumours. Over 40% of all cases of retinoblastoma are hereditary and conform to an autosomal dominant pattern of inheritance. Such tumours tend to appear at an earlier age than the sporadic, non-hereditary type and there is an increased incidence of multiple primaries. Tumours develop when both normal copies of the relevant gene are mutated or deleted, (Knudson, 1978). Draper *et al.* (1992) estimated the risk of retinoblastoma in sibs of affected patients. For the five families in which one child has unilateral retinoblastoma and a second a different type of tumour there was no family history of the disease; these sib pairs could result from unrecognised familial retinoblastoma, since it is known that a wide variety of tumours may occur in individuals carrying the retinoblastoma gene (Sanders *et al.*, 1989; Eng *et al.*, 1993). Alternatively some or all of these sib pairs may have arisen purely by chance as there are in the registry around 950 cases of presumed non-hereditary retinoblastoma and one might expect about three malignant tumours among their siblings simply on the basis of the normal population rate for these conditions.

For Wilms' tumour, Knudson and Strong (1972*a*) suggested a two-stage genetic model similar to that for retinoblastoma. In the six families in which two children had Wilms' tumour, only one of the children had bilateral tumours and the age distribution was similar to that for Wilms' tumour generally; this contrasts with the findings in retinoblastoma. In the United States National Wilms' Tumour Study, 37 out of 3442 cases (1.1%) were found to be familial; they had none of the features associated with genetic tumours (Breslow *et al.*, 1988).

In three families both children were diagnosed with neuroblastoma. Knudson and Strong (1972*b*) again postulated a two-stage genetic model for neuroblastoma similar to that for retinoblastoma. However, records from the CCRG show that the risk for a sibling of a neuroblastoma patient of developing the same cancer is of the order of 1 per 1000; this figure implies that only a very small proportion of cases are transmitted from a parent, i.e. that either the proportion of parents with germ-cell mutations is low (because the mutation rate is low or because survival of such potential parents is low) or that the manifestation rate (penetrance) of potential genetic cases is low.

Thus, for both Wilms' tumour and neuroblastoma the hereditary element appears to be much smaller than originally suggested by Knudson and Strong.

A considerable number of case reports has been published relating to childhood cancer in twins, but there are only two or three population-based studies from which concordance rates can be derived; even these yield estimates only for leukaemia. The NRCT contains a total of about 800 twins, of which about 240 are monozygotic. Thus, the great majority of twin pairs are not concordant for childhood cancer. For leukaemia there is a high concordance rate in monozygous twins, as suggested by the data in Table II, for cases diagnosed at an early age. For these cases the proband concordance rate, i.e. the risk to the co-twin, may be as high as 25%. In many, or perhaps most, of these cases the concordance appears to arise not from genetic similarity but from an *in utero* transfer of transformed cells from one fetus to the other (Chaganti *et al.*, 1979; Ford *et al.*, 1993). For hereditary retinoblastoma the proband concordance rate for monozygous twins can be directly calculated from the accepted penetrance of 90% as being 90–100%, depending on whether one assumes that the probabilities of the twins being affected are independent or, on the other hand, that if one is affected the other is more likely to be affected also. For other diagnostic groups there is no adequate evidence on which to base an estimate, although it appears likely that there is an increased risk for monozygous co-twins of affected children.

Children with Down's syndrome have 10–20 times the usual risk of leukaemia (Miller, 1963). In the NRCT 291 patients have been identified with Down's syndrome, although the diagnosis was not routinely verified by chromosome studies; the great majority, 277 (95%), of these patients were diagnosed with leukaemia. The family in this study that included one child with Down's syndrome and fibrosarcoma and a second child with osteosarcoma and congenital defects may represent an unidentified syndrome.

Down's and some other syndromes found in families in this study – hypospadias, gonadal dysgenesis, XYY syndrome and Ollier's disease – are known to be associated with cancer (McKusick, 1992). Sickle-cell anaemia and Ehlers–Danlos syndrome were noted in two other families. These are among autosomal dominant phenotypes listed by McKusick, but have not previously been linked with neoplasms.

A genetic element is clearly present in about a third of the 'sib pair' families in this study. For the remainder no clear pattern emerges; some may be caused by an unknown genetic predisposition, and some by a shared environmental exposure; some are undoubtedly due to chance. Diagnosis of cancer in three or more siblings almost certainly indicates some genetic susceptibility or immunological deficiency.

The main conclusion to be drawn from this study is that, except for cases associated with known genetic disease, the risk of cancer in the sibs of affected children is small: the current estimate of a doubling of the usual risk, i.e. from about 1 in 600 to 1 in 300, seems to be consistent with the results presented here. The main exception to this is the considerably higher risk of leukaemia for monozygous co-twins of children with leukaemia.

**Acknowledgements**
We thank the consultants, particularly members of the UK Children's Cancer Study Group, family doctors, the Office of Population Censuses and Surveys, the Information and Statistics Division of the Common Services Agency of the Scottish Health Service, the Registrar General for Scotland and regional cancer registries who provided notifications of cancers and deaths. We are especially grateful to the parents of children with cancer, who provided much of the information on which this paper is based. We thank Mr C.A. Stiller for comments and advice and Mrs E.M. Roberts for secretarial and other help. We are very grateful to Dr Leo Kinlea for suggested improvements to the paper. The Childhood Cancer Research Group is supported by the Department of Health and the Scottish Home and Health Department.

### References

BAIRD PN, PRITCHARD J AND COWELL JK. (1994). Molecular genetic analysis of chromosome 11p in familial Wilms' tumour. *Br. J. Cancer*, **69**, 1072-1077.

BITHELL JF AND STEWART A. (1975). Pre-natal irradiation and childhood malignancy. A review of British data from the Oxford survey. *Br. J. Cancer*, **31**, 271-287.

BRESLOW N, BECKWITH JB, CIOL M AND SHARPLES K. (1988). Age distribution of Wilms' tumor: Report from the national Wilms' tumor study. *Cancer Res.*, **48**, 1653-1657.

CHAGANTI RSK, MILLER DR, MEYERS PA AND GERMAN J. (1979). Cytogenic evidence of the intrauterine origin of acute leukemia in monozygotic twins. *N. Engl. J. Med.*, **300**, 1032-1034.

DRAPER GJ, HEAF MM AND KINNIER WILSON LM. (1977). Occurrence of childhood cancers among sibs and estimation of familial risks. *J. Med. Genet.*, **14**, 81-90.

DRAPER GJ, SANDERS BM, BROWNBILL PA, HAWKINS MM. (1992). Patterns of risk of hereditary retinoblastoma and applications to genetic counselling. *Br. J. Cancer*, **66**, 211-219.

ENG C, LI FP, ABRAMSON DH, ELLSWORTH RM, WONG FL, GOLDMAN MB, SEDDON J, TARBELL N AND BOICE JD JR (1993). Mortality from second tumors among long-term survivors of retinoblastoma. *J. Natl Cancer Inst.*, **85**, 1121-1128.

FORD AM, RIDGE SA, CABRERA ME, MAHMOUD H, STEEL CM, CHAN LC AND GREAVES M (1993). In utero rearrangements in the trithorax-related oncogene in infant leukaemias. *Nature*, **363**, 358-360.

GREEN DM. (1986). Childhood cancer in siblings. *Pediatr. Hematol. Oncol.*, **3**, 229-239.

KNUDSON AG. (1978). Retinoblastoma: a prototypic hereditary neoplasm. *Semin. Oncol.*, **5**, 57.

KNUDSON AG AND STRONG LC. (1972*a*). Mutation and cancer: A model for Wilms' tumour of the kidney. *J. Natl Cancer Inst.*, **48**, 313-324.

KNUDSON AG AND STRONG LC. (1972*b*). Mutation and cancer: neuroblastoma and pheochromocytoma. *Am. J. Hum. Genet.*, **24**, 514-532.

LI FP AND FRAUMENI JF. (1969). Soft-tissue sarcomas, breast cancer, and other neoplasms. A familial syndrome? *Ann. Intern. Med.*, **71**, 747-752.

LI FP, TUCKER MA AND FRAUMENI JF. (1976). Childhood cancer in sibs. *J. Pediatr.*, **88**, 419-423.

LI FP, FRAUMENI JF, MULVIHILL JJ, BLATTNER WA, DREYFUS MG, TUCKER MA AND MILLER RW (1988). A cancer family syndrome in twenty-four kindreds. *Cancer Res.*, **48**, 5358-5362.

MCKUSICK VA. (1992). *Mendelian Inheritance in Man. Catalogs of Autosomal Dominant, Autosomal Recessive, and X-Linked Phenotypes, Volume 1.* The John Hopkins University Press: London.

MANN JR, CORKERY JJ, FISHER HWJ, CAMERON AH, MAYEROVA A, WOLF U, KENNAUGHS AA AND WOOLEY V (1983). The X linked form of XY gonadal dysgenesis with a high incidence of gonadal germ cell tumours: clinical and genetic studies. *J. Med. Genet.*, **20**, 264-270.

MILLER RW. (1963). Down's syndrome (Mongolism), other congenital malformations and cancers among the sibs of leukemic children. *N. Engl. J. Med.*, **268**, 393-401.

MILLER RW. (1971). Deaths from childhood leukemia and solid tumors among twins and other sibs in the United States, 1960-67. *J. Natl Cancer Inst.*, **46**, 203-209.

MULVIHILL JJ. (1982). Ecogenetic origins of cancer in the young: Environmental and genetic determinants. In *Cancer in the Young*. Levine A.S. (ed.) pp. 13-27. Masson: New York.

SANDERS BM, JAY M, DRAPER GJ AND ROBERTS EM. (1989). Non-ocular cancer in relatives of retinoblastoma patients. *Br. J. Cancer*, **60**, 358-365.

STEWART A, WEBB J AND HEWITT D. (1958). A survey of childhood malignancies. *Br. Med. J.*, **11**, 1495-1508.

STILLER CA, CHESSELLS JM AND FITCHETT MF. (1994). Neurofibromatosis and childhood leukaemia/lymphoma: a population based UK CCSG study. *Br. J. Cancer.*, **70**, 969-972.

STILLER CA, ALLEN MB AND EATOCK EM. (1995). Childhood cancer in Britain: The national registry of childhood tumours and incidence rates 1978-87 *Eur. J. Cancer*, **31A**, 2028-2034.